# Integration of *vanHAX* downstream of a ribosomal RNA operon restores vancomycin resistance in a susceptible *Enterococcus faecium* strain

**DOI:** 10.1038/s44259-023-00017-0

**Published:** 2024-01-16

**Authors:** Ross S. McInnes, Ann E. Snaith, Steven J. Dunn, Maria Papangeli, Katherine J. Hardy, Abid Hussain, Willem van Schaik

**Affiliations:** 1 Institute of Microbiology and Infection, University of Birmingham, Birmingham, B15 2TT UK; 2 Biodiscovery Institute, National Biofilms Innovation Centre and School of Life Sciences, University of Nottingham, Nottingham, NG7 2RD UK; 3 Birmingham Public Health Laboratory, UK Health Security Agency, Birmingham, B9 5SS UK

**Keywords:** Bacterial genetics, Antibiotics, Bacterial infection

## Abstract

During the genomic characterisation of *Enterococcus faecium* strains (*n* = 39) collected in a haematology ward, we identified an isolate (OI25), which contained *vanA-*type vancomycin resistance genes but was phenotypically susceptible to vancomycin. OI25 could revert to resistance when cultured in the presence of vancomycin and was thus considered to be vancomycin-variable. Long-read sequencing was used to identify structural variations within the vancomycin resistance region of OI25 and to uncover its resistance reversion mechanism. We found that OI25 has a reduced ability to positively regulate expression of the *vanHAX* genes in the presence of vancomycin, which was associated with the insertion of an IS*6*-family element within the promoter region and the first 50 bp of the *vanR* gene. The vancomycin-resistant revertant isolates constitutively expressed *vanHAX* genes at levels up to 36,000-fold greater than OI25 via co-transcription with a ribosomal RNA operon. The vancomycin-resistant revertants did not exhibit a significant growth defect. During VRE outbreaks, attention should be paid to contemporaneous vancomycin-susceptible strains as these may carry silent vancomycin resistance genes that can be activated through genomic rearrangements.

## Introduction

*Enterococcus faecium* is a Gram-positive bacterium that is a commensal of the human gastrointestinal tract^1^. However, it is also an opportunistic pathogen that can cause bacteriaemia, endocarditis and urinary tract infections in immunocompromised hosts^2^. Genomic studies have revealed that the vast majority of clinical infections are caused by a phylogenetically defined cluster of *E. faecium* strains, which was termed clade A1^1^. *E. faecium* infections are difficult to treat as they are often resistant to aminoglycoside, fluoroquinolone, β-lactam, and glycopeptide drugs^3^.

Vancomycin is a bactericidal glycopeptide antibiotic that targets peptidoglycan of the bacterial cell wall^4^. Resistance to vancomycin is conferred by clusters of genes which replace the terminal D-alanyl D-alanine motif of the lipid II stem peptide with a D-alanyl D-lactate or D-alanyl D-serine motif, thereby greatly reducing the binding affinity of vancomycin^5^. There are currently ten known gene clusters that confer resistance to vancomycin in *E. faecium*, but the *vanA* and *vanB*-type clusters are the most prevalent^6,7^. Vancomycin resistance gene clusters are generally carried on mobile genetic elements, of which the transposon Tn*1546* and the integrative and conjugative element Tn*1549* encode *vanA*- and *vanB-*type resistance, respectively. These elements can be integrated into plasmids and chromosomes^8,9^.

An increasing number of *E. faecium* strains are being identified that contain the gene clusters required for vancomycin resistance but are phenotypically susceptible^10–12^. These strains are known as vancomycin-variable *E. faecium* (VVE)^13^. The mechanisms which lead to the susceptibility of these isolates are varied. Full or partial deletion of genes within the vancomycin resistance gene cluster is common, including in the regulatory genes *vanR*-*vanS*, or the D-alanyl D-alanine dipeptidase gene *vanX*^13–16^, as well as deletions in promoter sites and integration of insertion sequence (IS) elements into the promoter regions of vancomycin resistance genes^17,18^. Vancomycin-variable isolates are of particular concern in the treatment of patients as these isolates can rapidly revert to the resistant phenotype under vancomycin selection, which may, in turn, lead to treatment failure.

Here we investigate an outbreak of vancomycin resistant *Enterococcus faecium* in a haematology ward within a UK hospital. Within the outbreak we identified a vancomycin-variable isolate that was able to rapidly revert to a vancomycin-resistant phenotype under low-level vancomycin selection and we uncovered both the cause of its susceptibility and the mechanism by which it could revert to a vancomycin resistant phenotype.

## Results

### Genome sequence analysis revealed a multi-clonal, nosocomial VRE outbreak

*Enterococcus faecium* strains were isolated from a haematology ward in a hospital in Birmingham (United Kingdom) over a 2-year period (2016-2017) of increased vancomycin resistant *E. faecium* (VRE) bacteraemia. A total of 39 *E. faecium* isolates were collected from 24 patients. Thirty-four of the isolates were from blood culture samples and five were isolated from rectal screening swabs of patients. Twenty-six isolates were phenotypically resistant to vancomycin and 13 were phenotypically susceptible (Table S1).

**Fig. 1:**
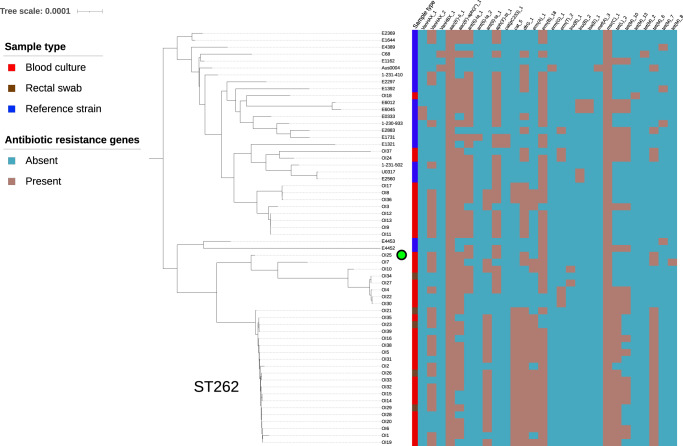
Maximum likelihood core genome phylogenetic tree of the clinical *E. faecium* isolates and representative clade A1 isolates. Metadata includes the sample type (Blood culture, Rectal swab or Reference strain) and the presence or absence of antibiotic resistance genes. The scale bar indicates the number of substitutions per site. The green circle indicates the VVE isolate OI25.

Phylogenetic analysis of the clinical *E. faecium* isolates uncovered a complex population of isolates belonging to clade A1 (Fig. S1). Eight different sequence types (ST262, ST80, ST1478, ST780, ST117, ST203, ST412 and ST787) were isolated on the ward during the period of the outbreak. A dominant ST262 clone that was present in 13 patients was the likely driver of the outbreak within the haematology ward. While all isolates could be assigned to clade A1, they were distinct from the clade A1 reference isolates (Fig. 1). The outbreak isolates contained a large repertoire of antibiotic resistance genes (Fig. 1). Aminoglycoside resistance was common among the isolates, with isolates carrying between two and five aminoglycoside resistance genes. All outbreak isolates carried the *E. faecium* intrinsic aminoglycoside resistance *aac(6’)-Ii* gene^19^ and 33 of the 39 isolates carried the *aac(6’)-aph(2”)* gene. Erythromycin resistance genes were also found in all outbreak isolates: *erm(B)* was the most common macrolide resistance gene and was found in 33 of the isolates. Tetracycline resistance genes were found in 29 isolates, including *tet(L)* and four different alleles of *tet(M)*. Vancomycin resistance was widespread in the isolates with 25 out of 39 isolates carrying vancomycin resistance genes, all of which were the *vanA*-type. It was noted that isolate OI25 was phenotypically susceptible to vancomycin but carried the *vanHAX* genes necessary to confer phenotypic resistance, which suggested that it was a vancomycin-variable *Enterococcus faecium* (VVE) isolate. To confirm the result of the VITEK 2 susceptibility testing, the MIC of vancomycin for *E. faecium* isolate OI25 was determined by broth microdilution. Isolate OI25 had a vancomycin MIC of 1 μg/ml, which is below the EUCAST clinical breakpoint of 4 μg/ml, confirming that this isolate was indeed susceptible to vancomycin despite carrying the genes required for phenotypic resistance to vancomycin.

### OI25 had a reduced ability to positively regulate expression of *vanHAX* in the presence of vancomycin

**Fig. 2:**
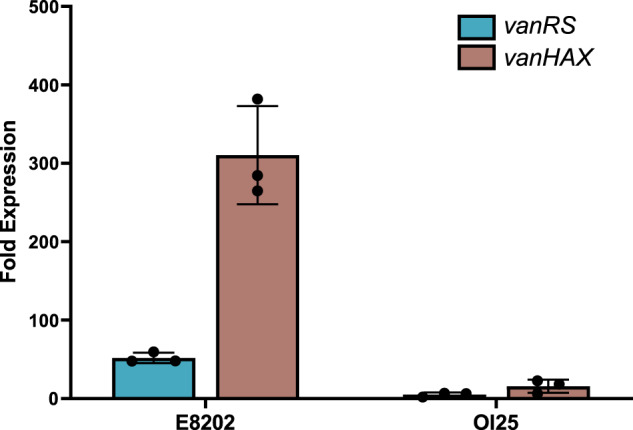
Expression of *vanHAX* and *vanRS* in *E. faecium* E8202 and the VVE isolate OI25. RT-qPCR analysis of the change in expression of the vancomycin resistance gene operons *vanHAX* and *vanRS* of E8202 and the VVE isolate OI25 after exposure to 8 μg/ml vancomycin. Expression data was normalised to the reference gene *tufA*. Experiments were carried out with biological triplicates and technical duplicates. Error bars represent standard deviation.

RT-qPCR analysis was used to compare the transcriptional response of the vancomycin resistance operons *vanRS* and *vanHAX* in isolate OI25 to that of a vancomycin-resistant isolate (E8202) when exposed to 8 µg/ml vancomycin (Fig. 2). Expression of the *vanHAX* operon increased 310-fold in isolate E8202 when exposed to vancomycin but increased only 16-fold in isolate OI25. Similarly, upon exposure to vancomycin, expression of the *vanRS* genes increased 52-fold in the wildtype VRE isolate but only 5-fold in isolate OI25. This demonstrated that the susceptibility of isolate OI25 to vancomycin was due to its reduced ability to increase gene expression of the *vanHAX* operon in the presence of vancomycin.

### An IS*6*-family element disrupted the *vanR* gene and its promoter in OI25

**Fig. 3:**
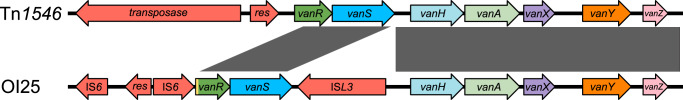
Alignment of the vancomycin resistance region of *E. faecium* isolate OI25 against the vancomycin resistance region of the prototypical Tn*1546* transposon. The Tn*1546* sequence was obtained from NCBI Genbank (accession number: M97297.1). Grey boxes represent regions which are identical between isolates. The yellow box represents the deletion in *vanR*.

A genome assembly, incorporating both long- and short reads, of isolate OI25 was generated to analyse the vancomycin resistance region, in order to identify a possible mechanism that abolished vancomycin resistance in this isolate. The vancomycin resistance genes of OI25 were located on a completely assembled 36,651 bp plasmid. Compared to the prototypical Tn*1546* transposon (GenBank: M97297.1), isolate OI25 had an insertion of an IS*L3*-family element between the *vanS* and *vanH* genes (Fig. 3). However, this insertion did not occur within the previously characterised promoter region of *vanH* and thus did not disrupt the two VanR binding sites upstream of *vanH*^20^. OI25 also had an insertion of an IS*6*-family element within the promoter region and the first 50 bp of the *vanR* gene, which disrupted the *vanR* open reading frame. It was likely that this inactivated *vanR*, thus preventing activation of the *vanHAX* genes in the presence of vancomycin, leading to the vancomycin-susceptible phenotype of OI25.

### Reversion to a high-level vancomycin resistant phenotype

Isolate OI25 was exposed to 8 μg/ml vancomycin to investigate whether it could revert to a vancomycin-resistant phenotype in the presence of a low concentration of vancomycin. Growth was observed within the OI25 culture after 48 h. Two isolates taken from this culture had a vancomycin MIC of 512 μg/ml, thus showing that isolate OI25 could revert to a vancomycin resistant phenotype under vancomycin selection. The frequency of reversion of OI25 from a vancomycin-susceptible to vancomycin-resistant phenotype was determined to be 1.0 × 10^−7^ ± 0.8 × 10^−7^ (standard deviation, *n* = 3) resistant/susceptible colony forming units after 48 h of culture with 8 μg/ml vancomycin.

### Insertion of vancomycin resistance genes downstream of a ribosomal RNA operon led to a vancomycin-resistant phenotype

**Fig. 4:**
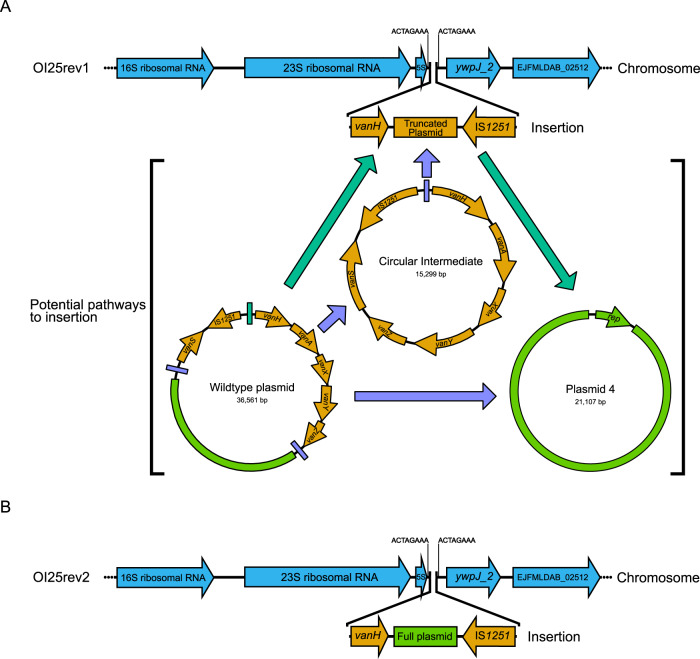
Mechanisms of VVE reversion to vancomycin resistance. **A** Insertion of the vancomycin resistance plasmid into the chromosome of OI25rev1 and the possible intermediate stages in the insertion. **B** Insertion of the vancomycin resistance genes into the chromosome of OI25rev2.

Variant calling between OI25 and the revertant isolates (OI25rev1 and OI25rev2) was implemented to identify differences that may have been responsible for the switch from a vancomycin-susceptible to resistant phenotype. Compared to OI25, OI25rev1 had a non-synonymous P69L mutation in a copy of IS*1062* and an intergenic A > AT insertion. OI25rev2 contained the same two variants as OI25rev1, but also had a T416K non-synonymous mutation in a hypothetical gene and an insertion of a short fragment of DNA (TTTTATCTACATCGTTTTGTCTG) within an intergenic region. As the variants identified in the revertant isolates were not obviously linked to the restoration of vancomycin resistance, complete genome assemblies of OI25 and its revertant isolates were generated to identify structural changes in the genome which could have caused phenotypic reversion. In both OI25 revertant isolates (OI25rev1 and OI25rev2), we observed similar genomic rearrangements, i.e. the insertion of the vancomycin resistance genes into the chromosome, with the *vanHAX* operon becoming inserted immediately downstream of a ribosomal RNA operon, while *vanRS* was located at the opposite end of the insertion (Fig. 4). In isolate OI25rev1 a 15,299-bp fragment of the plasmid DNA was integrated in the chromosome while a 21,107-bp plasmid remained (Fig. 4A), whereas in isolate OI25rev2 the entire plasmid was integrated into the chromosome (Fig. 4B). It could not be ascertained whether in isolate OI25rev1 the whole plasmid was integrated and then excised leaving the vancomycin resistance genes behind in the chromosome (Fig. 4A; green arrows) or whether the vancomycin resistance genes were excised and formed an intermediate mobile genetic element that was then integrated into the chromosome (Fig. 4A; purple arrows). In both isolates there was an 8-bp target site duplication (ACTAGAAA) surrounding the DNA inserted into the chromosome that is consistent with the action of an IS element.

### Substantial, constitutive upregulation of *vanHAX* expression in revertant isolates

As the insertion of the plasmid DNA into the chromosome did not restore the *vanR* gene it was hypothesised that the *vanHAX* genes were instead being constitutively expressed. To determine whether the expression of the *vanHAX* operon had changed at this new locus, RT-qPCR was used to compare the expression of the *vanHAX* and *vanRS* operons in the revertant isolates compared to OI25. Although the insertions that occurred in both revertant isolates were different, the change in expression of the *vanHAX* and *vanRS* operons was similar. In the absence of vancomycin, the expression of the *vanHAX* operon in the two revertant strains OI25rev1 and OI25rev2 was on average ( ± standard deviation) 2.7 × 10^4^ ± 1.1 × 10^4^-fold and 3.6 × 10^4^ ± 1.3 × 10^4^-fold greater than in OI25. The expression of the *vanRS* operon was also 39.4 ± 22.8-fold (OI25rev1) and 34.2 ± 16.3-fold (OI25rev2) higher in the revertants, compared to OI25, despite the continued disruption of the *vanR* gene. This demonstrated that the revertant isolates were expressing the *vanHAX* genes needed to confer resistance to vancomycin, even in the absence of vancomycin.

The genomic insertion site was inspected in both the revertant and parent isolates to determine a mechanism behind the constitutive expression of the *vanHAX* genes. In the parental OI25 strain, a putative rho-independent terminator of the ribosomal RNA operon was uncovered (Fig. S2). The chromosomal insertion of plasmid DNA in isolates OI25rev1 and OI25rev2 occurred 27 bp downstream of the 5S rRNA gene stop codon. This insertion occurred approximately halfway through the putative rho-independent terminator leading to the disruption of its secondary structure. It was hypothesised that disruption of the rho-independent terminator could lead to the co-transcription of the ribosomal RNA genes and the *vanHAX* genes.

**Fig. 5:**
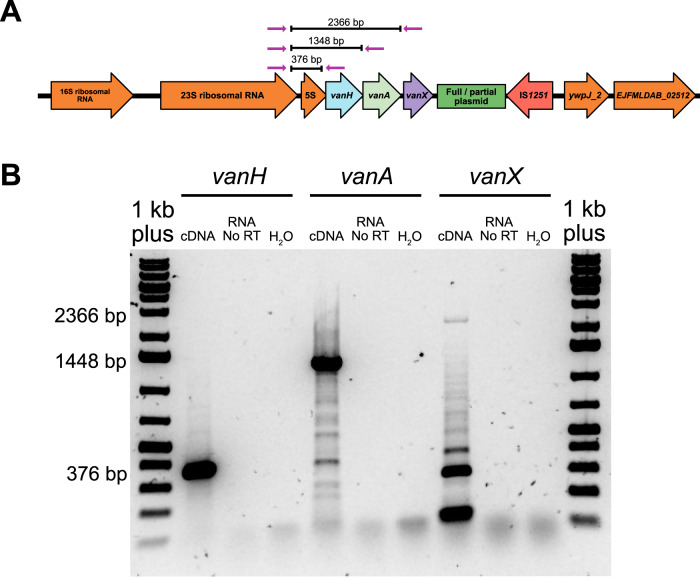
RT-PCR on the rRNA-*vanHAX* junction in OI25rev2. **A** Schematic showing the expected amplicon sizes. **B** 1% agarose gel showing the amplicons with the expected products sizes from panel (**A**) indicated for the RT-PCR reactions between the 23S rRNA gene and *vanH* (376 bp), *vanA* (1348 bp) and *vanX* (2366 bp). Ladder: GeneRuler 1 kb Plus (Thermo Scientific). RT Reverse Transcriptase.

To determine whether the *vanHAX* operon was co-transcribed with the upstream ribosomal RNA gene operon, RNA was reverse transcribed from isolates OI25rev1 and OI25rev2 and PCR was performed across the rRNA - *vanHAX* operon junction. Three PCR reactions were performed on the cDNA each of which spanned from the 23S ribosomal RNA gene into the *vanH*, *vanA* and *vanX* genes (Figs. 5, S3). PCR amplicons of the expected lengths were present for all three gene which confirmed that the *vanHAX* genes were indeed co-transcribed with the ribosomal RNA genes.

### Vancomycin-resistant revertant isolates did not show a significant growth defect and were phenotypically stable

**Fig. 6:**
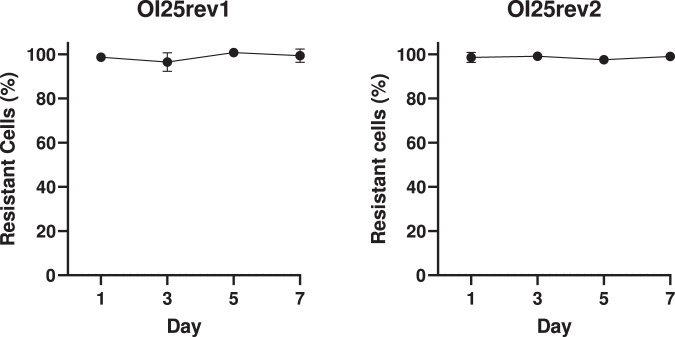
Stability of vancomycin resistance in revertant isolates OI25rev1 and OI25rev2. Stability of the vancomycin resistance phenotype in the absence of vancomycin as measured by the percentage of resistant cells within the total population over a seven-day period. At day 0, cultures were inoculated with a single colony and stability was assayed on subsequent days by determining colony forming units on BHI agar with and without vancomycin. Points represent the mean value and error bars represent the standard error of the mean. Experiments were carried out with biological and technical triplicates.

It was hypothesised that co-transcription of the vancomycin resistance genes with the ribosomal RNA genes would impose a high fitness cost in the revertant isolates. However, when the wildtype isolate OI25 and its revertants were grown in the absence of vancomycin selection (Fig. S4), µ_max_ of isolate OI25 (1.7 h^−1^) was not significantly different to that of OI25rev1 (1.7 h^−1^, Kruskal-Wallis, *P* > 0.99) or OI25rev2 (1.8 h^−1^, Kruskal-Wallis, *P* = 0.11). Similarly, the maximum growth reached by OI25rev1 (A_600_ 0.29) and OI25rev2 (A_600_ 0.25) was lower, but not significantly different, from that of OI25 (A_600_ 0.36; Kruskal-Wallis versus OI25rev1 *P* = 0.22 and versus OI25rev2 *P* = 0.08). Despite the vancomycin resistance genes being transcribed at a high level in the revertant isolates, this did not impose a significant fitness cost. To further assess the fitness cost of the chromosomal insertion of *vanHAX*, the stability of the vancomycin resistance phenotype was measured in the absence of vancomycin. Over a seven-day period of culturing, the percentage of resistant cells remained stable, and above 95% of the total population, for both OI25rev1 and OI25rev2 (Fig. 6).

## Discussion

The present study aimed to investigate a VRE outbreak in a haematology ward. The isolates in this study belonged to 8 different sequence types within the hospital-associated clade A1^1^. The major clone driving the outbreak belonged to ST262, with the presence of highly related ST262 isolates in 13 different patients suggesting spread within the ward. ST262 has previously been associated with the hospital environment in the UK and Europe but has not thus far been identified as a prominent driver of a VRE outbreak^21–23^. Other isolates belonged to ST80 which has been linked to VRE outbreaks in Ireland and Sweden^24,25^.

An isolate (OI25) belonging to ST787 was identified that was genotypically resistant to vancomycin but phenotypically susceptible. Long-read sequencing uncovered multiple IS element insertions into the vancomycin resistance regions compared to the wildtype transposon Tn*1546*^8^. An IS*L3* family element was inserted between the *vanS* and *vanH* genes. This insertion likely did not contribute to the susceptibility of the isolate as it occurred outside of the promoter region and an identical insertion has been found in other isolates which maintain a resistant phenotype^26^. There was also a further insertion of an IS*6* family element into the promoter region and first 50 bp of the *vanR* gene. This insertion was unique among global isolates, but a similar vancomycin-variable *E. faecium* isolate has been described, which also contained an insertion of an IS*6* family element that deleted the first 55 bp of the *vanR* gene^17^. As the insertion of the IS*6* element occurred within the *vanR* gene and its promoter region it was likely that isolate OI25 could not respond to vancomycin which was subsequently confirmed by RT-qPCR.

Although vancomycin-variable enterococci are phenotypically susceptible to vancomycin, these isolates can revert to a resistant phenotype under antibiotic selection. Several mechanisms have been uncovered including the use of alternative promoters driving *vanHAX* expression and increases in plasmid copy numbers^17^, and gene duplication events^27^. Exposure of isolate OI25 to 8 µg/ml vancomycin led to a reversion of the isolate to high-level vancomycin resistance. The reversion rate of 1.0 × 10^−7^ was in line with other VVE isolates described in the literature^17,28^. Bacterial numbers achieved during *E. faecium* colonisation of the intestinal tract are such that the in vivo reversion of isolate OI25 to a vancomycin resistance phenotype is possible^29^. Variant calling was performed on two revertant isolates against parent VVE isolate OI25 to identify differences between the susceptible and resistant isolates. A small number of variants were identified in both revertant isolates, but were either found within intergenic regions or IS elements, and thus were not thought to have contributed to the phenotypic reversion. Long-read sequencing of the two revertant isolates uncovered that the vancomycin resistance genes *vanH*, *vanA* and *vanX* had become inserted into the chromosome directly downstream of a ribosomal RNA operon. This insertion caused a disruption of the rho-independent terminator of the operon and led to the co-transcription of the vancomycin resistance genes in a constitutive manner. The native high-level expression of the ribosomal RNA genes led to a significant upregulation in the *vanHAX* genes^30^. The presence of an 8 bp target site duplication and an IS*L3* family element at the 3’ end of the inserted DNA suggested an IS mediated rearrangement of the DNA through a currently uncharacterised mechanism^31^. Despite the high-level expression of the *vanHAX* operon, the chromosomal insertion did not incur a large fitness cost and proved to be stable over an extended period of time in the absence of vancomycin. This suggests that the vancomycin resistant phenotype would be maintained within a patient even after the withdrawal of vancomycin treatment. We have not been able to find examples of similar integration events of resistance genes immediately downstream of rRNA operons and thus believe this may represent a newly discovered mechanism by which phenotypic resistance can be restored upon the loss of transcriptional control of resistance gene expression.

Our findings highlight the diversity of mechanisms that enable VVE isolates to revert to their resistant state. While vancomycin-variable *E. faecium* typically make up a small percentage of the *E. faecium* strains isolated within the clinical environment, they have, in places, become the dominant clone^11^. As VVE isolates become more common in the hospital environment it may be of interest to include whole genome long-read sequencing in surveillance of vancomycin-resistant enterococci to rapidly identify strains that are phenotypically susceptible to vancomycin but can potentially revert to high-level vancomycin resistance.

## Methods

### Collection and isolation of *Enterococcus faecium*

*Enterococcus faecium* strains were isolated from a haematology ward in a hospital in Birmingham (United Kingdom) over a 2-year period (2016-2017). Thirty-nine isolates were collected from 24 patients by blood culture and rectal screening. The blood culture samples were taken from febrile patients, while rectal screening samples were collected from all patients on the ward. Only vancomycin-resistant rectal screening isolates from patients with VSE bacteraemia were included in this study. Bacteria were initially isolated on Columbia CNA agar (Oxoid) plates and were confirmed as *Enterococcus faecium* by MALDI-TOF (Bruker). The *vanA*^+^ isolate *E. faecium* E8202 was used as a control for gene expression in Tn*1546*^32^.

### Short- and long-read sequencing

DNA extraction and whole genome shotgun sequencing (WGS) using Illumina technology was carried out by MicrobesNG (http://www.microbesng.com). Isolates were lysed by suspending in TE buffer (Invitrogen) containing 0.1 mg/ml lysozyme (Thermo Scientific) and 0.1 mg/ml RNase A (ITW Reagents), the suspension was incubated at 37 °C for 25 min. Proteinase K (VWR Chemicals) and SDS (Sigma Aldrich) were added to a final concentration of 0.1 mg/ml and 0.5% v/v respectively and incubated for a further 5 min at 65 °C. DNA was purified using an equal volume of SPRI beads and resuspended in EB buffer (Qiagen). DNA libraries were prepared using the Nextera XT Library Prep Kit (Illumina) and pooled libraries were sequenced on an Illumina HiSeq instrument using a 250 bp paired-end protocol.

High molecular weight DNA was extracted from isolate OI25 and its revertants using the Monarch® HMW DNA Extraction Kit for Tissue (New England Biolabs) according to the manufacturer’s protocol with the addition of 50 μg/ml lysozyme (Sigma Aldrich) to weaken the cell wall during the lysis step. The DNA libraries were prepared using the ligation sequencing kit SQK-LSK109 (Oxford Nanopore Technologies) and sequenced on the MinION platform (Oxford Nanopore Technologies) using a R9.4.1 flowcell (Oxford Nanopore Technologies).

### Genome assembly

Adaptors were removed from the short-read data and quality trimmed using fastp v.0.20.1^33^. Reads less than 50 bp were discarded and a sliding window quality cut-off of 15 was used. The short-read data was then assembled using shovill v.1.0.4 (https://github.com/tseemann/shovill) using the default parameters. Hybrid assemblies were created by Unicycler v.0.4.8^34^ using both short and long reads, Unicycler was run using the default parameters. Both the short-read and hybrid assemblies were annotated using PROKKA v.1.14.6^35^.

### Phylogenetic analysis

Core genome alignments were created with Panaroo v.1.2.2^36^ using –clean-mode strict. We included reference genomes (*n* = 72) from Lebreton et al.^1^, which span the diversity of the species *Enterococcus faecium* and specifically the hospital associated clade A1 (*n* = 21), to contextualise the genomes of strains that were isolated in this study. Phylogenetic trees were created from the core genome alignments using RAxML v.8.1.15^37^ implementing the GTRGAMMA substitution model with 100 bootstraps. Recombination was removed from the trees using ClonalFrameML v.1.12^38^. Trees were midpoint rooted and visualised using iTOL v.5^39^. Isolates were typed with PubMLST^40^ using mlst v.2.18.0 (https://github.com/tseemann/mlst).

### Identification of antibiotic resistance determinants in *E. faecium* genomes

Antibiotic resistance genes were identified in the *E. faecium* isolates by querying the short-read assemblies against the ResFinder database^41^ using ABRicate v.0.9.8 (https://github.com/tseemann/abricate). A minimum identity and coverage cut-off of 95 and 50%, respectively, was used to determine that the antibiotic resistance genes were present.

### Antibiotic susceptibility testing

All outbreak isolates were tested for their antibiotic susceptibility using the VITEK2 system (Biomérieux). A subset of the isolates was also tested using the broth microdilution method^42^ and interpreted with the EUCAST breakpoints. Assays were carried out in biological triplicate and the mode of the minimum inhibitory concentration was recorded. *E. faecium* E745 was used as a positive control in all assays^43^.

### Variant calling

Snippy v.4.6.0 (https://github.com/tseemann/snippy) was used to identify sequence variants in the revertant genomes by mapping their Illumina short reads to the Genbank annotation file of isolate OI25 that was previously generated by PROKKA v.1.14.6^35^.

### VVE reversion rate and stability of the revertant isolates

A colony of isolate OI25 was inoculated into 5 ml of Brain Heart Infusion (BHI) broth (VWR) and grown at 37 °C for 16 h with shaking (200 rpm). The culture was then diluted 1:100 into 5 ml of BHI broth containing 8 µg/ml vancomycin. The culture was grown at 37 °C (200 rpm) and observed every 24 h for growth. When growth was observed, the culture was diluted 10^6^-fold and 100 µl was spread onto BHI agar plates containing 8 µg/ml vancomycin. Two colonies were picked from the plate and stored for further analysis. To determine the rate of reversion from susceptible to resistant, a culture of OI25 grown at 37 °C for 16 h with shaking (200 rpm) was diluted in a 10-fold series and spread on BHI agar plates with and without 8 µg/ml vancomycin. The plates were incubated at 37 °C for 48 h, and the number of colonies was counted on both sets of plates. The reversion rate was calculated as the ratio of resistant colonies to the total population. Stability of the resistant phenotype was determined by culturing OI25 in the absence of vancomycin for 7 days. Each day the culture was diluted 1:100 into 5 ml of BHI broth. On days 1, 3, 5 and 7 the culture was diluted in a 10-fold series and spread on BHI agar plates with and without 8 µg/ml vancomycin, and the percentage of resistant cells to total cells was determined.

### Reverse transcription-quantitative polymerase chain reaction (RT-qPCR) and Reverse transcription polymerase chain reaction (RT-PCR)

RNA was extracted from cultures collected in mid-log phase (OD_600_ = 0.5) and cultures that had been exposed to 8 μg/ml vancomycin at mid-log phase for 1 h, using the Monarch® Total RNA Miniprep Kit (New England Biolabs). Residual DNA was removed by treating the RNA with TURBO DNase^TM^ (Invitrogen). cDNA was synthesised from the total RNA using the Maxima First Strand cDNA Synthesis Kit for RT-qPCR (Thermo Scientific). qPCR was carried out using PrimeTime® Gene Expression Master Mix (2X) (Integrated DNA Technologies (IDT)) and PrimeTime® qPCR Assays (20X) (IDT), which contained the forward primer, reverse primer and probe, for the *vanRS* and *vanHAX* operons, and the *tufA* reference gene (Table S2). The qPCR reaction was performed in a QuantStudio 1 Real-Time PCR system (Applied Biosystems^TM^) with the following programme: 95 °C for 3 min, followed by 40 cycles of 95 °C for 15 s and 60 °C for 1 min. Fold expression was calculated using the Livak method relative to the reference gene *tufA*^44^.

The cDNA of isolate OI25rev1 and OI25rev2 was also used to perform RT-PCR assays across the rRNA-*vanHAX* junction. RT-PCR reactions were carried out using DreamTaq 2x Mastermix (Thermo Fisher Scientific) and forward and reverse primers that bridged between the 23S rRNA gene and the *vanH*, *vanA* and *vanX* genes (Table S3). The reactions were performed in a Mastercycler Pro Thermal Cycler (Eppendorf) with the following programme: 95 °C for 3 min, followed by 30 cycles of 95 °C for 30 s, 50 °C for 30 s and 72 °C for 2 min, followed by a final incubation at 72 °C for 10 min. A reaction with a sample from which reverse transcriptase was omitted was used to control for residual DNA.

### Terminator analysis

The rho-independent terminator of the ribosomal RNA gene operon was identified in isolate OI25 by analysing 100 nucleotides downstream of the 5S rRNA gene stop codon via RNAfold Web Server^45^. The output was then manually inspected to identify the typical A-tail, loop, T-tail structure of a rho-independent terminator^46^.

### Fitness evaluation

Bacterial fitness was evaluated by comparing the maximum growth rate (µ_max_; h^−1^) and maximum growth (maximum A_600_) of the revertant isolates compared to isolate OI25. Bacterial cultures were grown for 16 h at 37 °C in BHI broth, diluted 1:1000 in BHI broth and added to a clear flat-bottom 96-well plate. Wells were included that contained only BHI broth to control for changes in A_600_ not caused by bacterial growth. The 96-well plate was incubated at 37 °C with agitation (240 rpm) for 16 h, absorbance measurements (600 nm) were taken at 10-min intervals using a Spark microplate reader (TECAN). The experiment was carried out in biological and technical triplicates. Maximum growth rate and maximum growth were determined using the R package Growthcurver v.0.3.1^47^.

### Statistical analyses

Tests for determining statistical significance were performed as described in the text and implemented in GraphPad Prism v.9.4.1.

### Ethics

This study did not require ethical approval as it was part of a hospital infection control investigation into a local outbreak.

### Reporting summary

Further information on research design is available in the Nature Research Reporting Summary linked to this article.

## Supplementary information

Supplemental Materials

Reporting Summary

## Data Availability

Raw sequencing reads have been deposited in the European Nucleotide Archive under accession number PRJEB57409. Hybrid assemblies of the VVE strain OI25 and its revertants have been deposited in Genbank under accession numbers GCA_947511065.1, GCA_947511075.1 and GCA_947510805.1.

## Code availability

All software was used as described in the cited literature unless stated otherwise in the methods section.
