# Supplementary material for: Integration of vanHAX downstream of a ribosomal RNA operon restores vancomycin resistance in a susceptible Enterococcus faecium strain
Source: NPJ Antimicrob Resist. 2024 Jan 16;2:2. doi: 10.1038/s44259-023-00017-0 (PMC11702846; doi:10.1038/s44259-023-00017-0)
Supplement: Supplementary file 1 — Supplemental Materials [file 44259_2023_17_MOESM1_ESM.pdf]

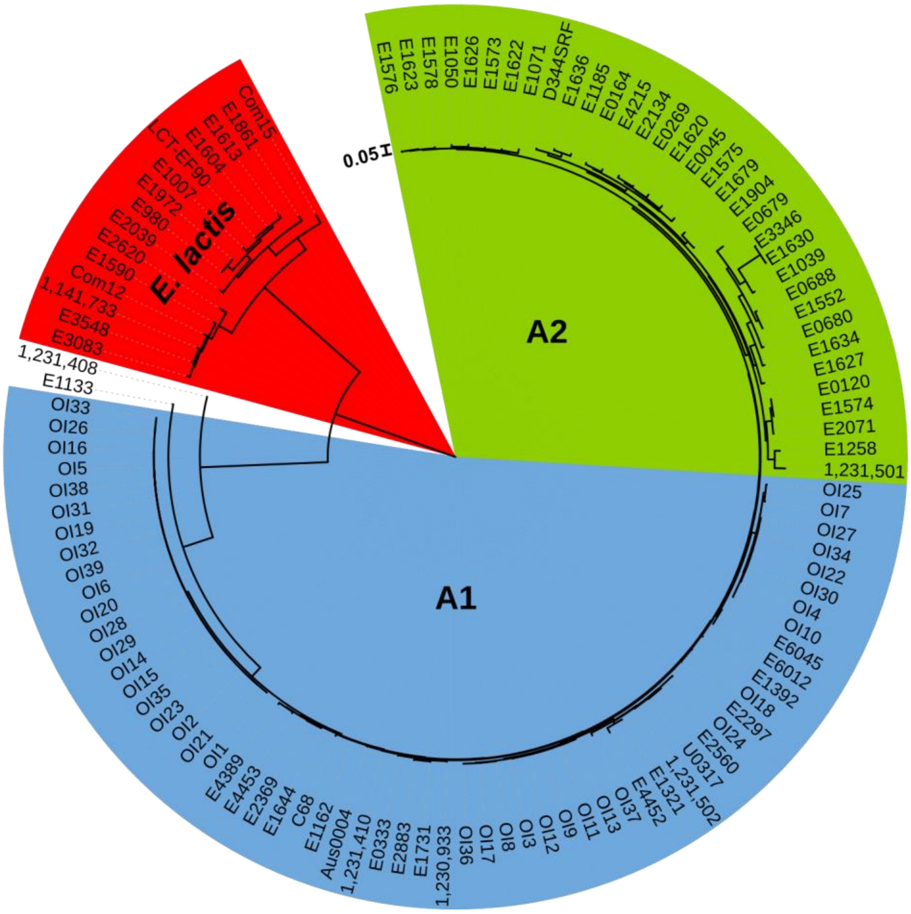

Figure S1: Maximum likelihood core genome phylogenetic tree of the clinical *E. faecium* isolates and representative genomes from across the species *E. faecium*. The major clades of *E. faecium* have been highlighted: clade A1 (blue), clade A2 (green) and *E. lactis* (previously clade B; red). The scale bar indicates the number of substitutions per site.

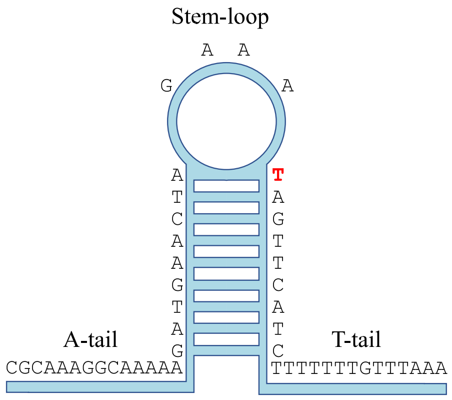

Figure S2: Putative rho-independent terminator of the ribosomal RNA operon in isolate OI25. A schematic representation of the putative rho-independent terminator of the ribosomal RNA operon. The nucleotide at which the insertion occurs in the revertant isolates has been highlighted in red.

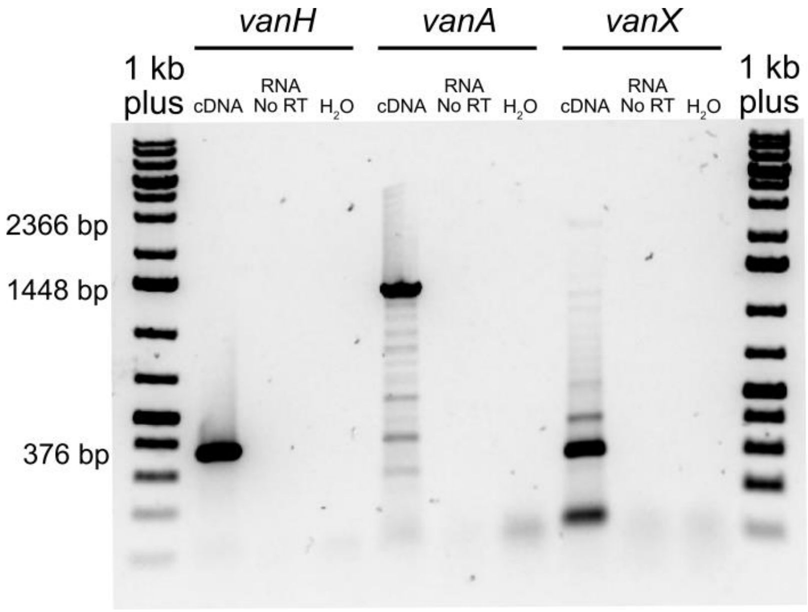

Figure S3: RT-PCR on the rRNA-*vanHAX* junction in OI25rev1. 1% agarose gel showing the amplicons with the expected products sizes for the RT-PCR reactions between the 23S rRNA gene and *vanH* (376 bp), *vanA* (1348 bp) and *vanX* (2366 bp). Ladder: GeneRuler 1kb Plus (Thermo Scientific). RT: Reverse Transcriptase.

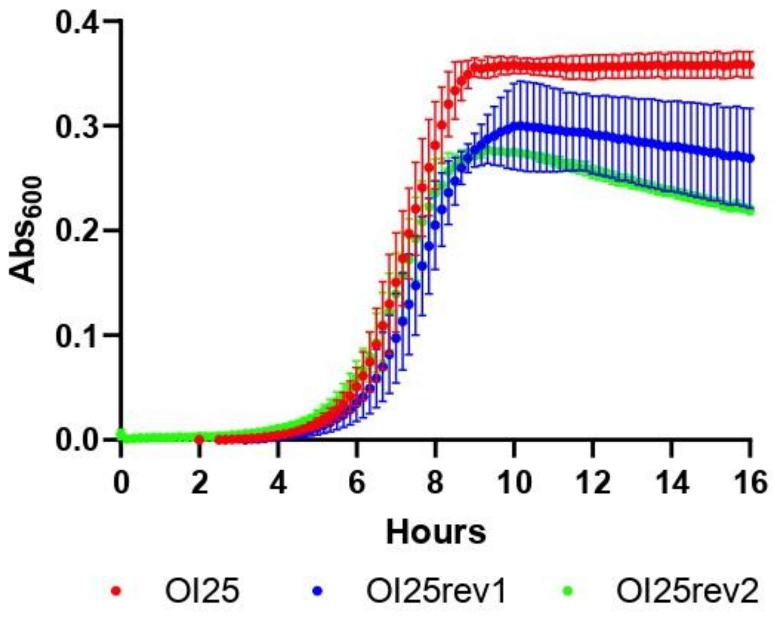

Figure S4: Growth of OI25 and its revertant isolates. Growth kinetics of OI25 and revertants OI25rev1 and OI25rev2. Abs_{600}: Absorbance measured at 600 nm. The mean data is presented, and the error bars represent the standard deviation of the mean.

Table S1: Metadata of the outbreak isolates

Isolate name	Patient	Isolation Month	Sample type	MLST	Vancomycin phenotype	Vancomycin genotype
OI1	23	Mar-16	Blood Culture	262	Resistant	Resistant
OI2	1	Apr-16	Blood Culture	262	Resistant	Resistant
OI3	2	May-16	Blood Culture	1478	Resistant	Resistant
OI4	3	May-16	Blood Culture	80	Resistant	Resistant
OI5	4	Jun-16	Blood Culture	262	Resistant	Resistant
OI6	6	Feb-17	Blood Culture	262	Resistant	Susceptible
OI7	7	Feb-17	Blood Culture	80	Resistant	Resistant
OI8	8	Feb-17	Blood Culture	780	Resistant	Resistant
OI9	9	Apr-17	Blood Culture	1478	Resistant	Resistant
OI10	10	Apr-17	Blood Culture	80	Resistant	Resistant
OI11	11	May-17	Blood Culture	1478	Resistant	Resistant
OI12	12	May-17	Blood Culture	1478	Resistant	Resistant
OI13	13	May-17	Blood Culture	1478	Resistant	Resistant
OI14	14	May-17	Blood Culture	262	Resistant	Resistant
OI15	14	Jun-17	Blood Culture	262	Resistant	Resistant
OI16	15	Jun-17	Blood Culture	262	Resistant	Resistant
OI17	24	Jul-17	Blood Culture	780	Susceptible	Susceptible
OI18	7	Feb-17	Blood Culture	117	Susceptible	Susceptible
OI19	5	Dec-16	Blood Culture	262	Resistant	Resistant
OI20	17	Aug-17	Blood Culture	262	Susceptible	Susceptible
OI21	22	Nov-17	Rectal Swab	262	Resistant	Resistant
OI22	18	Nov-17	Blood Culture	80	Susceptible	Susceptible
OI23	18	Nov-17	Rectal Swab	262	Resistant	Resistant
OI24	13	May-17	Blood Culture	412	Susceptible	Susceptible
OI25	1	Dec-16	Blood Culture	787	Susceptible	Resistant
OI26	19	Oct-17	Rectal Swab	262	Resistant	Resistant
OI27	20	Nov-17	Blood Culture	80	Susceptible	Susceptible
OI28	21	Aug-17	Blood Culture	262	Susceptible	Susceptible
OI29	21	Sep-17	Rectal Swab	262	Resistant	Resistant
OI30	22	Aug-17	Blood Culture	80	Susceptible	Susceptible
OI31	4	Jun-16	Blood Culture	262	Susceptible	Susceptible
OI32	6	Feb-17	Blood Culture	262	Resistant	Resistant
OI33	19	Oct-17	Blood Culture	262	Susceptible	Susceptible
OI34	20	Nov-17	Rectal Swab	80	Resistant	Susceptible
OI35	16	Jul-17	Blood Culture	262	Resistant	Resistant
OI36	17	Aug-17	Blood Culture	780	Resistant	Resistant
OI37	12	May-17	Blood Culture	203	Susceptible	Susceptible
OI38	4	Jun-16	Blood Culture	262	Resistant	Resistant
OI39	6	Feb-17	Blood Culture	262	Susceptible	Susceptible
OI25rev1	NA	Jan-20	Laboratory evolved	787	Resistant	Resistant
OI25rev2	NA	Jan-20	Laboratory evolved	787	Resistant	Resistant

NA = Not applicable

Table S2: RT-qPCR primers to measure expression of *vanHAX* and *vanRS* operons.

**Primer name**	**Sequence (5’ -> 3’)**	**Description**
tufA_fwd	GGTGACGATGTTCCTGTAGTT	PrimeTime qPCR Probe assay targeting the *tufA* gene in *E. faecium*.
tufA_probe	TGAAAGCTCTAGAAGGCGACGCTT	
tufa_reverse	CGTTCTGGAGTTGGGATGTATT	
vanHAX_fwd	ATATAAAGCGCTCGGCTGTAG	PrimeTime qPCR Probe assay targeting the *vanHAX* operon in *E.faecium*.
vanHAX_probe	TAACGGCCGCATTGTACTGAACGA	
vanHAX_rev	TGAAACCGGGCAGAGTATTG	
vanRS_fwd	AAGCTGGCCGAACAAAGA	PrimeTime qPCR Probe assay targeting the *vanRS* operon in *E.faecium*.
vanRS_probe	ACGTTGTTATGTACTTGGCGCACG	
vanRS_rev	CGTCAAGCAGGCTCAAATAAC	

Table S3: RT-PCR primers to test for co-transcription of rRNA genes and *vanHAX* genes.

**Prime name**	**Sequence (5’ -> 3’)**	**Description**
Junc_fwd	CGGACTGATACTAATCGATCG	Primer used to check for junction between rRNA and *vanHAX* genes in cDNA.
Junc_vanH_rev	GGAATGCATCTGCCTCATC	Primer used to check for junction between rRNA and *vanH* gene in cDNA.
Junc_vanA_rev	AGATTTTACCGATACGTCATGC	Primer used to check for junction between rRNA and *vanA* gene in cDNA.
Junc_vanX_rev	CAACGAACACCGTGTACTAT	Primer used to check for junction between rRNA and *vanX* gene in cDNA.
